# Research on Evaluation of Green Smart Building Based on Improved AHP-FCE Method

**DOI:** 10.1155/2021/5485671

**Published:** 2021-11-26

**Authors:** Song Xu, Yao Sun

**Affiliations:** School of Economics and Management, Anhui Jianzhu University, Hefei, Anhui, China

## Abstract

With the accelerated pace of urbanization, green buildings and green smart buildings gradually come into people's vision and are highly valued by all sectors of society on the premise of meeting sustainable development strategy. Firstly, this paper selects 7 first-level index factors and 20 second-level index factors to establish the green smart building evaluation system. Secondly, this paper uses the analytic hierarchy process-fuzzy comprehensive evaluation (AHP-FCE) method to determine the weight of each secondary index. Finally, the feasibility of the evaluation system is verified by case analysis, and some suggestions on green smart building are put forward.

## 1. Introduction

A large number of buildings will be built in the construction of new urbanization. However, buildings are one of the largest energy consumers in the world. As people pay more and more attention to issues such as energy, environment, and sustainable development, the development of green smart architecture has become a new direction that conforms to the new urbanization construction. In the “Guiding Opinions on Accelerating the Establishment and Improvement of a Green Low-Carbon Circular Development Economic System” issued by China's State Council in February 2021, it is emphasized that green planning, green design, and green construction should be carried out in an all-round way; high-quality development and high-level protection should be promoted so as to ensure the realization of the goals of carbon peak and carbon neutrality [1]. In today's fast-developing construction industry, to achieve the goals of building energy conservation, environmental protection, and greenness and to provide humans with a safe, comfortable, and healthy production and living environment, the construction industry needs to shift from rapid development to high-quality development. Green smart building is a new-generation building incorporating BIM, GIS, Internet of Things, cloud computing, and other technologies. It saves resources and improves energy utilization while reducing environmental pollution and resource waste and has a great effect on alleviating the current energy shortage in my country. At present, there are relatively mature evaluation standards for the evaluation of green buildings, but there are few studies on the comprehensive evaluation of green smart buildings. Combining the smart building evaluation index factors, this paper tries to build a simple and clear green smart building evaluation system based on the green building evaluation system so as to enrich the new green building evaluation standards and promote the evaluation and development of green smart buildings.

## 2. Research Status

Arkin and Paciuk pointed out that intelligent buildings are increasingly using intelligent devices, materials, and sensors. Intelligent buildings should provide environments and means for the best use of buildings. They studied some contemporary intelligent buildings based on the level of system integration [2]. Green buildings are buildings related to resource efficiency, life cycle effects, and building performance; smart buildings with integrated building technology systems as the core are buildings related to building and operational efficiency, as well as enhanced management and occupant functions. Sinopoli has studied the commonalities between the two [3]. Runde and Fay pointed out that building automation requires a large number of smart devices, and modern building automation systems are composed of as many as thousands of components with many attributes and dependencies [4]. Robichaud and Anantatmula's research shows that by adding a team of professionals to the project, they can promote the completion of green building projects better and faster [5]. Chen and Huang suggested the establishment of an environmental health information management platform to provide residential users with a comfortable and healthy indoor environment [6]. Balta-Ozkan et al. defined an intelligent building as a residence equipped with a communication network, linking sensors, household appliances, and devices that can be remotely monitored, accessed, or controlled to provide services that respond to the needs of its residents. They studied the similarities and differences in the technical and economic driving factors and obstacles to the development of the smart home market in three European countries characterized by different policies and socioeconomic backgrounds [7]. Shaikh et al. conducted a comprehensive and important research on the most advanced intelligent control system for energy and comfort management of intelligent energy buildings [8]. Buckman et al. claimed in 2014 that intelligence can be used interchangeably with smart, and there is no obvious difference between the two [9]. Attoue et al. proposed the concept of smart buildings to use smart technology to reduce energy consumption and improve comfort and user satisfaction [10]. Research by To et al. found that building users tend to focus more on intelligent security systems, followed by intelligent and responsive fresh air supply, elevators, and escalators [11]. Ding and Fan pointed out that most green buildings certified by rating tools are mainly evaluated based on their design and construction. The life cycle of green buildings goes beyond these initial stages, and their full benefits become more apparent during the operation phase of the building [12]. Zhao et al. reviewed and analyzed 2,980 articles published from 2000 to 2016; the results show that green building research is concentrated in the fields of engineering, environmental science, ecology, and construction technology [13]. Apanaviciene et al. research and define the characteristics that smart buildings should meet in order to be compatible with the overall background of smart cities and introduce a new evaluation framework for smart buildings to integrate into smart cities [14]. Eini et al. proposed a real-time management system to control all aspects of smart buildings and proposed the system's performance specifications, design requirements, and operational constraints [15].

Long et al. [16] started from the concept of intelligent buildings and indoor ecological environment and introduced the use of passive methods such as energy-saving windows and building exterior sunshades and the use of active methods such as displacement ventilation and cold radiation ceilings to improve the indoor environment of smart buildings. After analyzing the concept and characteristics of green building and intelligent building as well as their development status at home and abroad, Yin et al. [17] put forward the harmonious and unified view of “human, building and nature” in order to achieve the purpose of saving energy and resources, harmless, pollution-free and recyclable, harmonious and sustainable development of society. Through a large number of investigations, combined with engineering construction practices, Duan [18] integrated a variety of green building evaluation systems to develop a green construction evaluation standard for construction projects. Wang and Zhou [19] studied in depth the green building evaluation system proposed by the American LEED company and the “Green Building Evaluation Standards” issued by China and combined the two standards for comparative analysis, then constructed a simple evaluation system using AHP method. Liu and Peng [20] based on the in-depth understanding of green building and real estate development, combined green building and real estate to build a green real estate development evaluation index system, adopted AHP-FCE method to establish a green real estate evaluation model, and combined with index weights put forward policy recommendations for realizing green real estate development. Xiong et al. [21] comparatively analyzed domestic and foreign green building evaluation systems, and on this basis, they built a green intelligent building evaluation system based on the 2014 version of green building evaluation standards, established a five-level evaluation standard, and determined the weights of evaluation indicators and a comprehensive evaluation model. Wang et al. [22, 23] analyzed and studied the influence of EBI, FCS, and AIOT technologies on the building automation system of modern green intelligent buildings. The application of these technologies further enhanced and improved the control level, use functions, and service efficiency of green intelligent buildings. These technologies lay the foundation for the real realization of the “green” and “intelligence” of buildings and create conditions for the further transformation of intelligent buildings into super-intelligent buildings and smart buildings.

Based on academic research at home and abroad, scholars have continuously studied green buildings and intelligent buildings. The evaluation objects focused on green intelligent buildings mainly include “four savings and one environmental protection,” intelligent equipment, technology, environment, materials, and management. These evaluation systems have laid the foundation for the development of green smart buildings. Under the policy background of green economy and sustainable development, we have established a green smart building evaluation system, including safety and durability, health and comfort, convenience of life, resource conservation, environmental livability, smart, innovation and characteristic indicators and then used the analytic hierarchy process-fuzzy comprehensive evaluation (AHP-FCE) method to determine the weight of each secondary indicator and established a five-level evaluation standard.

## 3. Modeling Steps of Improved Analytic Hierarchy Process-Fuzzy Comprehensive Evaluation (AHP-FCE) Method

### 3.1. Establish a Set of Evaluation Indicators

We need to build a judging evaluation index system for the goal. Generally speaking, the fuzzy comprehensive discriminant model includes three levels of indicators, namely, the target level, the criterion level, and the plan level factor set. The evaluation object *U* is a collection of evaluation indicators, which is hierarchical. The first-level indicators can be established as (*U*_*i*_),  *i*=1,2,3, ⋯, *n*, so the index system is

(1) $$\begin{array}{c} U=\left(U_{1},U_{2},\cdots ,U_{n}\right). \end{array}$$

The secondary indicators can be established as (*U*_*ij*_), so

(2) $$\begin{array}{c} U_{i}=\left(U_{i1},U_{i2},\cdots ,U_{iN_{i}}\right),\;\;i=1,2,\cdots ,n, \end{array}$$

*N*_*i*_ is the number of secondary indicators included in *U*_*i*_.

### 3.2. Establish Evaluation Grade

(3) $$\begin{array}{c} V=\left(V_{1},V_{2},\cdots ,V_{K}\right), \end{array}$$

where *V*_*j*_( *j*=1,2, ⋯, *K*) is the classification of different grades.

### 3.3. Construct Fuzzy Relation Matrix

#### 3.3.1. Construct Judgment Matrix *U∗*

(4) $$\begin{array}{c} U*=\left[\begin{array}{cccc} u_{11} & u_{12} & \cdots & u_{1n}\\ u_{21} & u_{22} & \cdots & u_{2n}\\ \vdots & \vdots & \ddots & \vdots \\ u_{n1} & u_{n2} & \cdots & u_{nn} \end{array}\right], \end{array}$$

where $u_{ij}=\left\{\begin{array}{cc} 0, & \text{ the }i\text{ factor }U_{i}\text{ is not as important as the }j\text{ factor }U_{j},\\ 1, & \text{ the }i\text{ factor }U_{i}\text{ and the }j\text{ factor }U_{j}\text{ are equally important,}\\ 2, & \text{ the }i\text{ factor }U_{i}\text{ is more important then the }j\text{ factor }U_{j}, \end{array}\right\}$.

For the fuzzy relation matrix, also known as the membership matrix, it is necessary to establish not only the comment set, but also the membership set of grade factors. In this way, after quantitative analysis, the specific position of each factor that may affect the evaluation object in the grade can be determined so as to form the fuzzy relation matrix *P*:

(5) $$\begin{array}{c} P_{U_{k}}\left(i,j\right)=p_{ij}^{k},\;\;k=1,2,\cdots ,n,\;i=1,2,\cdots ,N_{k}\;,\;j=1,2,\cdots ,K, \end{array}$$

where *p*_*ij*_^*k*^=*v*_*ijk*_/*M*, where *v*_*ijk*_  is the number of experts who believe that *U*_*ki*_ is affiliated to *V*_*j*_ among all experts. *M* is the total number of experts.

### 3.4. Calculate Weight Using Improved AHP Method

AHP analytic hierarchy process is a multiobjective decision analysis method that combines qualitative and quantitative analysis methods. The improved analytic hierarchy process in this article is based on the traditional analytic hierarchy process, draws lessons from the methods in Ba's academic achievements [24], and makes changes in the strategy of constructing the judgment matrix. The previous nine-scale method is replaced by a more concise three-scale method, which makes it easier for experts to understand. Judging and scoring is more intuitive. The improved AHP method improves the accuracy of judgment, and the consistency check step can be omitted after using the optimal transfer matrix, which reduces the computational workload [25, 26].

Then, we solve the element *h*_*ij*_ in the judgment matrix *H*:

(6) $$\begin{array}{c} h_{ij}=\left\{\begin{array}{cc} \frac{\left(r_{i}-r_{j}\right)\left(k_{m}-1\right)}{r_{\max}-r_{\min}}+1, & \;r_{i}\geq r_{j},\\ {\left[\frac{\left(r_{j}-r_{i}\right)\left(k_{m}-1\right)}{r_{\max}-r_{\min}}+1\right]}^{-1} & \;r_{i}<r_{j}, \end{array}\right\}, \end{array}$$

where *r*_*i*_=∑_*j*=1_^*n*^*u*_*ij*_,  *r*_max_=max(*r*_*i*_), *k*_*m*_=*r*_max_/*r*_min_.

Let *E*=[*e*_*ij*_]_*n*×*n*_, where

(7) $$\begin{array}{c} q_{ij}=\log\;h_{ij},\\ d_{ij}=\frac{1}{n}\sum_{k=1}^{n}\left(q_{ik}-q_{jk}\right),\\ \;e_{ij}=10^{d_{ij}}. \end{array}$$

Calculate *M*_*i*_, the product of each row element of matrix *E* constructed above, then calculate its *n*th root. The result is as follows:

(8) $$\begin{array}{c} W_{i}=\sqrt[{n}]{M_{i}}=\sqrt[{n}]{\Pi e_{ij}}. \end{array}$$

Normalize the vector *W* to get *W*_*i*_′: *W*_*i*_′=(*w*_*i*_/∑_*i*=1_^*n*^*w*_*i*_); finally, we can get the weight vector *W* of *n* elements:

(9) $$\begin{array}{c} W=\left(W_{1},W_{2},\cdots ,W_{n}\right). \end{array}$$

## 4. Green Smart Building Evaluation Index System Based on Improved AHP-FCE Method

To build a more systematic and comprehensive evaluation system for green smart building projects, it is necessary to select the first and second indicators and the corresponding scoring rules, and the indicators should be relatively independent so as to avoid the appearance of redundant and miscellaneous indicators. At the same time, in order to facilitate the understanding of calculations and applications, the construction of the index system should also be simple and easy to implement. Following the principles of systemicity, dynamics, and relative independence, combined with China's latest “Green Building Evaluation Standard” (GB/T50378-2019) and the group standard “Smart Building Evaluation Standard” issued by China Building Energy Conservation Association in 2021, we have built an evaluation index system for green smart buildings in Table 1.

## 5. Empirical Analysis

Xiang'an Zhengrong Mansion is located at the intersection of Shamei Road and Xiang'an South Road. It was built by XM Zhengpeng Real Estate Co., Ltd. The total construction area of the project is 114,307.13 square meters, covering an area of 27,595.52 square meters, the greening rate is 30%, and the plot ratio is 2.8. The planned properties include commercial streets, landscape gardens, and basketball courts. The project is surrounded by Xiangshan Park and Shamei Park. The environment is beautiful, and it is close to the subway entrance and exit, making travel very convenient.

### 5.1. Building Evaluation System Based on Improved AHP-FCE Model

Next, we use the improved AHP-FCE method to comprehensively evaluate the green wisdom project level of Xiang'an Zhengrong Mansion in combination with the 7 primary index factors and 20 secondary index factors listed in Table 1.

### 5.2. Construction of Judgment Matrix and Single-Layer Weight Calculation

According to the green smart building evaluation index system established in Table 1, the hierarchical structure is constructed by combining the interrelationships between the indicators. Experts from the green smart building and real estate industries are invited to compare and score each factor. A judgment matrix is constructed, and the corresponding weights are calculated. The results are as follows:

(10) $$\begin{array}{c} U*=\left[\begin{array}{ccccccc} 1 & 2 & 2 & 0 & 2 & 0 & 2\\ 0 & 1 & 2 & 0 & 1 & 0 & 2\\ 0 & 0 & 1 & 0 & 0 & 0 & 2\\ 2 & 2 & 2 & 1 & 2 & 1 & 2\\ 0 & 1 & 2 & 0 & 1 & 0 & 2\\ 2 & 2 & 2 & 1 & 2 & 1 & 2\\ 0 & 0 & 0 & 0 & 0 & 0 & 1 \end{array}\right]. \end{array}$$

Calculate according to the steps of the improved fuzzy comprehensive evaluation method, and get the weights of each criterion layer (first-level indicators):

(11) $$\begin{array}{c} W_{U}=\left(W_{U_{1}},W_{U_{2}},W_{U_{3}},W_{U_{4}},W_{U_{5}},W_{U_{6}},W_{U_{7}}\right)\;,\\ \;=\left(0.1481,\;0.0607,\;0.0253,\;0.3451,\;0.0607,\;0.3451,\;0.0151\right).\; \end{array}$$

Using the same method and principle, construct the judgment matrix of the index layer (secondary indicators) against the criterion layer:

Safety and durability indicators *U*_1_=(*U*_11_, *U*_12_), $U_{1}*=\left[\begin{array}{cc} 1 & 2\\ 0 & 1 \end{array}\right]$.

Health and comfort indicators *U*_2_=(*U*_21_, *U*_22_, *U*_23_, *U*_24_), $U_{2}*=\left[\begin{array}{cccc} 1 & 2 & 0 & 0\\ 0 & 1 & 0 & 0\\ 2 & 2 & 1 & 0\\ 2 & 2 & 2 & 1 \end{array}\right]$.

Convenience of life indicators $U_{3}=\left(U_{31},U_{32},U_{33}\right),\;U_{3}*=\left[\begin{array}{ccc} 1 & 0 & 0\\ 2 & 1 & 2\\ 2 & 0 & 1 \end{array}\right]$.

Save resources indicators *U*_4_=(*U*_41_, *U*_42_, *U*_43_, *U*_44_), $U_{4}*=\left[\begin{array}{cccc} 1 & 0 & 0 & 0\\ 2 & 1 & 2 & 2\\ 2 & 0 & 1 & 2\\ 2 & 0 & 0 & 1 \end{array}\right]$.

Livable environment indicators *U*_5_=(*U*_51_, *U*_52_), $U_{5}*=\left[\begin{array}{cc} 1 & 2\\ 0 & 1 \end{array}\right]$.

Smart indicators *U*_6_=(*U*_61_, *U*_62_, *U*_63_), $U_{6}*=\left[\begin{array}{ccc} 1 & 0 & 0\\ 2 & 1 & 0\\ 2 & 2 & 1 \end{array}\right]$.

Innovation and characteristics indicators *U*_7_=(*U*_71_, *U*_72_), $U_{7}*=\left[\begin{array}{cc} 1 & 1\\ 1 & 1 \end{array}\right]$.

Calculate according to the improved method, and get the weight of each indicators layer (secondary indicators):

Safety and durability index weight *W*_*U*_1__=(0.7500,  0.2500).

Health and comfort index weight *W*_*U*_2__=(0.1178,  0.0550,  0.2634,  0.5638).

Convenience of life index weight *W*_*U*_3__=(0.1047,  0.6370,  0.2583).

Save resources index weight *W*_*U*_4__=(0.0550,  0.5638,  0.2634,  0.1178).

Livable environment index weight *W*_*U*_5__=(0.7500,  0.2500).

Smart index weight *W*_*U*_6__=(0.1047,  0.2583,  0.6370).

Innovation and characteristics index weight *W*_*U*_7__=(0.5000,  0.5000).

### 5.3. Calculation of the Composite Weight of Each Layer Element to the Target Layer

Through the above calculation and evaluation results, the weight of each indicator for comprehensive evaluation of green smart building project is obtained, as shown in Table 2.

**Figure 1:**
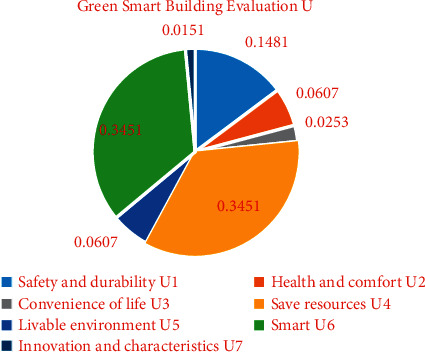
The weighting diagram of the criterion layer indicators.

**Figure 2:**
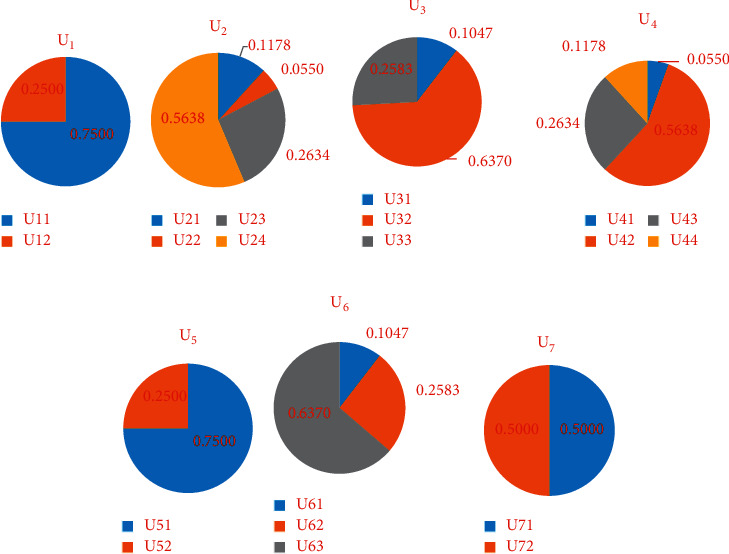
The weighting diagram of scheme layer indicators. (a) Weights of *U*_11_ – *U*_12_. (b) Weights of *U*_21_ – *U*_24_. (c) Weights of *U*_31_ – *U*_33._ (d) Weights of *U*_41_ – *U*_44_. (e) Weights of *U*_51_ – *U*_52_. (f) Weights of *U*_61_ – *U*_63._ (g) Weights of *U*_71_ – *U*_72_.

The weight distribution of indicators in Table 1 is shown in Figures 1 and 2. The main indicators that affect the evaluation of green smart buildings are save resources (*U*_4_, weight is 0.3451) and smart (*U*_6_, weight is 0.3451), followed by safety and durability (*U*_1_, weight is 0.1481). The main indicator that affects safety and durability (*U*_1_) is safety (*U*_11_, weight is 0.7500); the main indicator that affects health and comfort (*U*_2_) is indoor hot and humid environment (*U*_24_, weight is 0.5638); the main indicator that affects convenience of life (*U*_3_) is service facilities (*U*_32_, weight is 0.6370); the main indicator that affects save resources (*U*_4_) is energy-saving and energy utilization (*U*_42_, weight is 0.5638); the main indicator that affects livable environment (*U*_5_) is site ecology and landscape (*U*_51_, weight is 0.7500); the main indicator that affects smart (*U*_6_) is smart operation (*U*_63_, weight is 0.6370); the main indicators that affect innovation and characteristics (*U*_7_) are improvement and innovation (*U*_71_, weight is 0.5000) and characteristics (*U*_72_, weight is 0.5000).

**Figure 3:**
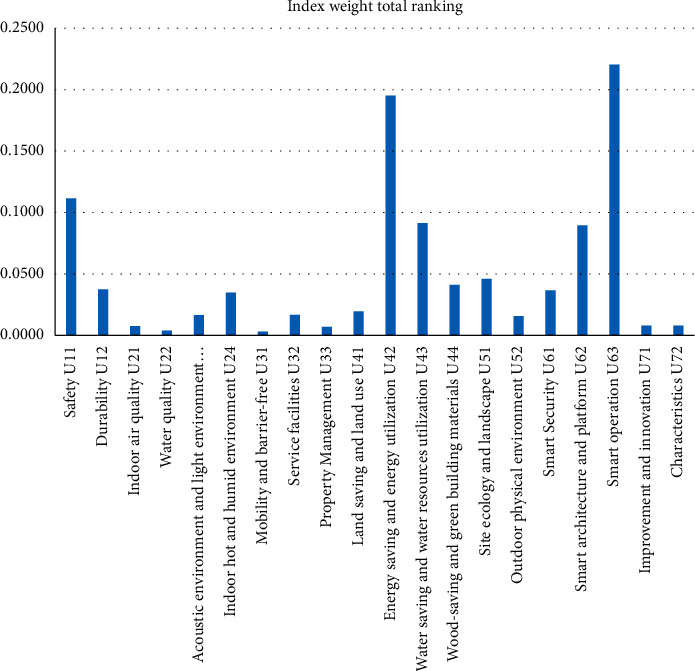
Comparison of weights of indicators.

The overall ranking of indicator weights is shown in Figure 3. Among all the impact indicators, the most important is smart operation (*U*_63_), followed by energy-saving and energy utilization (*U*_42_), followed by safety (*U*_11_), water-saving and water resources utilization (*U*_43_), and smart architecture and platform (*U*_62_).

### 5.4. Determine the Set of Evaluation Criteria

The evaluation standard set of green and smart building projects selects the five-star evaluation system in the “Smart Building Evaluation Standards,” which are one-star, two-star, three-star, four-star, and five-star. Use *V* to denote the set of evaluation criteria; then they are as follows:

(12) $$\begin{array}{c} V=\left\{V_{1},V_{2},V_{3},V_{4},V_{5}\right\},\\ =\left\{\text{one}-\text{star, two}-\text{star, three}-\text{star, four}-\text{star, five}-\text{star}\right\}\;,\\ =\left\{\left(0∼20\right],\;\left(20∼50\right],\left(50∼70\right]\;,\left(70∼90\right]\;,\;\left(90∼100\right]\right\}. \end{array}$$

### 5.5. Fuzzy Comprehensive Evaluation of Criterion Level

According to the actual situation of the project, this paper consulted a 10-member expert group composed of experts in the construction, environmental protection, and real estate industries by collecting relevant information and using questionnaire surveys and collected the expert group's review opinions on green and smart building projects. The fuzzy evaluation matrix is as follows:

Safety and durability index matrix:
  (13) $$\begin{array}{c} P_{U_{1}}=\left[\begin{array}{ccccc} 0.2 & 0.4 & 0.2 & 0.1 & 0.1\\ 0.2 & 0.5 & 0.2 & 0.1 & 0 \end{array}\right]. \end{array}$$
Health and comfort index matrix:
  (14) $$\begin{array}{c} P_{U_{2}}=\left[\begin{array}{ccccc} 0.1 & 0.6 & 0.1 & 0.1 & 0.1\\ 0.3 & 0.4 & 0.3 & 0 & 0\\ 0.4 & 0.6 & 0 & 0 & 0\\ 0.2 & 0.5 & 0.3 & 0 & 0 \end{array}\right]. \end{array}$$
Convenience of life index matrix:
  (15) $$\begin{array}{c} P_{U_{3}}\;=\left[\begin{array}{ccccc} 0.4 & 0.5 & 0.1 & 0 & 0\\ 0.1 & 0.5 & 0.4 & 0 & 0\\ 0.3 & 0.4 & 0.3 & 0 & 0 \end{array}\right]. \end{array}$$
   Save resources index matrix:
  (16) $$\begin{array}{c} P_{U_{4}}=\left[\begin{array}{ccccc} 0.1 & 0.5 & 0.2 & 0.1 & 0.1\\ 0.1 & 0.6 & 0.2 & 0.1 & 0\\ 0.3 & 0.4 & 0.3 & 0 & 0\\ 0.2 & 0.6 & 0.2 & 0 & 0 \end{array}\right]. \end{array}$$
Livable environment index matrix:
  (17) $$\begin{array}{c} P_{U_{5}}=\left[\begin{array}{ccccc} 0.1 & 0.5 & 0.3 & 0.1 & 0\\ 0.2 & 0.6 & 0.2 & 0 & 0 \end{array}\right]. \end{array}$$
Smart index matrix:
  (18) $$\begin{array}{c} P_{U_{6}}=\left[\begin{array}{ccccc} 0.2 & 0.5 & 0.1 & 0.2 & 0\\ 0.4 & 0.4 & 0.2 & 0 & 0\\ 0.2 & 0.6 & 0.2 & 0 & 0 \end{array}\right]. \end{array}$$
Innovation and characteristics index matrix:
  (19) $$\begin{array}{c} P_{U_{7}}=\left[\begin{array}{ccccc} 0.5 & 0.5 & 0 & 0 & 0\\ 0.3 & 0.6 & 0.1 & 0 & 0 \end{array}\right]. \end{array}$$

According to the steps of the improved AHP method, the calculated weight vector *W* of each evaluation index is established, the fuzzy evaluation matrix is established, and the comprehensive evaluation vector of the criterion layer (first-level indexes) is calculated according to the formula *Y*=*W* × *P*.

Comprehensive evaluation vector of safety and durability index:

(20) $$\begin{array}{c} Y_{U_{1}}\;=W_{U_{1}}\times P_{U_{1}}\\ =\left(0.7500,\;0.2500\right)\times \left[\begin{array}{ccccc} 0.2 & 0.4 & 0.2 & 0.1 & 0.1\\ 0.2 & 0.5 & 0.2 & 0.1 & 0 \end{array}\right]\\ =\left(0.2000,\;0.4250,\;0.2000,\;0.1000,\;0.0750\right). \end{array}$$

Comprehensive evaluation vector of health and comfort index:

(21) $$\begin{array}{c} \;Y_{U_{2}}=W_{U_{2}}\times P_{U_{2}}\\ =\left(0.1178,\;0.0550,\;0.2634,\;0.5638\right)\times \left[\begin{array}{ccccc} 0.1 & 0.6 & 0.1 & 0.1 & 0.1\\ 0.3 & 0.4 & 0.3 & 0 & 0\\ 0.4 & 0.6 & 0 & 0 & 0\\ 0.2 & 0.5 & 0.3 & 0 & 0 \end{array}\right]\\ \;=\left(0.2464,\;0.5326,\;0.1974,\;0.0118,\;0.0118\right). \end{array}$$

Comprehensive evaluation vector of convenience of life index:

(22) $$\begin{array}{c} \;Y_{U_{3}}=W_{U_{3}}\times P_{U_{3}}\\ =\left(0.1047,\;0.6370,\;0.2583\right)\times \left[\begin{array}{ccccc} 0.4 & 0.5 & 0.1 & 0 & 0\\ 0.1 & 0.5 & 0.4 & 0 & 0\\ 0.3 & 0.4 & 0.3 & 0 & 0 \end{array}\right]\\ \;=\left(0.1831,\;0.4742,\;0.3428,\;0.0000,\;0.0000\right). \end{array}$$

Comprehensive evaluation vector of save resources index:

(23) $$\begin{array}{c} Y_{U_{4}}=W_{U_{4}}\times P_{U_{4}}\\ \;=\left(0.0550,\;0.5638,\;0.2634,\;0.1178\right)\times \left[\begin{array}{ccccc} 0.1 & 0.5 & 0.2 & 0.1 & 0.1\\ 0.1 & 0.6 & 0.2 & 0.1 & 0\\ 0.3 & 0.4 & 0.3 & 0 & 0\\ 0.2 & 0.6 & 0.2 & 0 & 0 \end{array}\right]\\ \;=\left(0.1645,\;0.5418,\;0.2263,\;0.0619,\;0.0055\right). \end{array}$$

Comprehensive evaluation vector of livable environment index:

(24) $$\begin{array}{c} \;Y_{U_{5}}=W_{U_{5}}\times P_{U_{5}}\\ \;=\left(0.7500,\;0.2500\right)\times \left[\begin{array}{ccccc} 0.1 & 0.5 & 0.3 & 0.1 & 0\\ 0.2 & 0.6 & 0.2 & 0 & 0 \end{array}\right]\\ \;=\left(0.1250,\;0.5250,\;0.2750,\;0.0750,\;0.0000\right). \end{array}$$

Comprehensive evaluation vector of smart index:

(25) $$\begin{array}{c} Y_{U_{6}}=W_{U_{6}}\times P_{U_{6}}\\ \;=\left(0.1047,\;0.2583,\;0.6370\right)\times \left[\begin{array}{ccccc} 0.2 & 0.5 & 0.1 & 0.2 & 0\\ 0.4 & 0.4 & 0.2 & 0 & 0\\ 0.2 & 0.6 & 0.2 & 0 & 0 \end{array}\right]\\ \;=\left(0.2517,\;0.5379,\;0.1895,\;0.0209,\;0.0000\right). \end{array}$$

Comprehensive evaluation vector of innovation and characteristics index:

(26) $$\begin{array}{c} Y_{U_{7}}=W_{U_{7}}\times P_{U_{7}}\\ =\left(0.5000,\;0.5000\right)\times \left[\begin{array}{ccccc} 0.5 & 0.5 & 0 & 0 & 0\\ 0.3 & 0.6 & 0.1 & 0 & 0 \end{array}\right]\\ \;=\left(0.4000,\;0.5500,\;0.0500,\;0.0000,\;0.0000\right). \end{array}$$

### 5.6. Fuzzy Comprehensive Evaluation of Target Layer

Using the relevant calculation rules of the fuzzy comprehensive evaluation, and according to the calculation results of the fuzzy comprehensive evaluation of the criterion layer (first-level indexes), construct the target layer fuzzy evaluation matrix of this project; then, the target layer fuzzy evaluation matrix is

(27) $$\begin{array}{c} P_{U}=\left[\begin{array}{ccccc} 0.2000 & 0.4250 & 0.2000 & 0.1000 & 0.0750\\ 0.2464 & 0.5326 & 0.1974 & 0.0118 & 0.0118\\ 0.1831 & 0.4742 & 0.3428 & 0.0000 & 0.0000\\ 0.1645 & 0.5418 & 0.2263 & 0.0619 & 0.0055\\ 0.1250 & 0.5250 & 0.2750 & 0.0750 & 0.0000\\ 0.2517 & 0.5379 & 0.1895 & 0.0209 & 0.0000\\ 0.4000 & 0.5500 & 0.0500 & 0.0000 & 0.0000 \end{array}\right]. \end{array}$$

According to formula *Y*=*W* × *P*, the comprehensive evaluation vector of the target layer is

(28) $$\begin{array}{c} Y_{U}=W_{U}\times P_{U}\\ \;=\left(0.1481,\;0.0607,\;0.0253,\;0.3451,\;0.0607,\;0.3451,\;0.0151\right)\;\\ \times \left[\begin{array}{ccccc} 0.2000 & 0.4250 & 0.2000 & 0.1000 & 0.0750\\ 0.2464 & 0.5326 & 0.1974 & 0.0118 & 0.0118\\ 0.1831 & 0.4742 & 0.3428 & 0.0000 & 0.0000\\ 0.1645 & 0.5418 & 0.2263 & 0.0619 & 0.0055\\ 0.1250 & 0.5250 & 0.2750 & 0.0750 & 0.0000\\ 0.2517 & 0.5379 & 0.1895 & 0.0209 & 0.0000\\ 0.4000 & 0.5500 & 0.0500 & 0.0000 & 0.0000 \end{array}\right]\\ =\left(0.2064,\;0.5200,\;0.2112,\;0.0487,\;0.0137\right). \end{array}$$

According to the principle of the maximum degree of membership, the comprehensive evaluation level of the green smart building project can be determined. The maximum comprehensive evaluation value of the green intelligent building project in this case is 0.5200, which belongs to the *two* − *star* level of the set of evaluation criteria. Then, we use the formula *S*=*Y* × *G*^*T*^ to calculate the comprehensive evaluation value of the green smart building project and obtain the quantified comprehensive evaluation result, where the value of the quantified evaluation standard set *G* is the median value of the corresponding value in the evaluation standard set *V*. So, the quantified comprehensive score *S* is

(29) $$\begin{array}{c} S=Y_{U}\times G^{\text{T}}=\left(0.2064,\;0.5200,\;0.2112,\;0.0487,\;0.0137\right)\times {\left(10,\;35,\;60,\;80,\;95\right)}^{\text{T}}=38.1330. \end{array}$$

### 5.7. Analysis of Evaluation Results

Through the above calculations, it is shown that the project developed by XM Zhengpeng Real Estate Co., Ltd., is a two-star building. According to the quantified comprehensive evaluation calculation result, the comprehensive score of the overall evaluation of the project is corresponding to the two-star level. If you score according to the judging rules rules in Table 1, you can get consistent results. However, the judging rules' scoring method needs to determine the weight or value of the rules, which also increases the workload of the experts for scoring. The improved AHP-FCE method can reduce the corresponding workload and improve work efficiency.

## 6. Conclusions and Recommendations

From the analysis of the evaluation results, it can be seen that smart and green building sustainability have become the core of modern green buildings. The main indicators that have an impact on the development of green smart buildings include safety and durability indicators, health and comfort indicators, convenience of life indicators, save resources indicators, livable environment indicators, smart indicators, and innovation and characteristics indicators. Under the premise of these seven indicators, a fuzzy comprehensive evaluation model for green smart building projects was established, and this evaluation system was verified through corresponding cases, which further enriched the green smart building evaluation system.

In order to promote the implementation of my country's green and smart building strategy and improve the level of green economy development, the following points should be given priority: (1) Firstly, we should focus on save resources. Green smart buildings are the inevitable trend of future development. Scientific management and advanced green and clean environmental protection technologies should be used in their development so as to improve energy efficiency, reduce building energy consumption, and improve people's quality of life. Therefore, local governments should vigorously support the development of green buildings, further increase research on the development of green building products, and promulgate relevant policies for support and subsidies in order to accelerate the upgrading of the green and smart building industry. (2) Secondly, in terms of smart, it is necessary to make full use of the Internet of Things, 5G, big data, cloud computing, artificial intelligence, and other technologies to create an economical, safe, reliable, efficient, convenient, and green ecological living environment through automatic sensing, ubiquitous connection, timely transmission, and information integration. While strengthening the utilization of green and smart building resources, qualified enterprises should be encouraged to explore and innovate more advanced management systems and smart management. (3) In terms of safety and durability, attention should be paid to the safety and durability of buildings to avoid “fragile buildings.” Starting from the full life cycle of the building, improve the seismic performance of the building and the durability of structural components, and ensure the safety of people's lives.

Green smart buildings are developing rapidly. We should constantly learn from experience and adjust the direction in the course of its development so as to explore an optimal development path. In the context of carbon peaks and carbon neutrality, leading companies in green smart buildings should adhere to the green, environmentally friendly, and healthy production concepts and strive to explore zero-carbon buildings to provide a “green model” for the development of the industry.

## Figures and Tables

**Table 1:** Green smart building evaluation system.

Target layer	First-level indicators	Secondary indicators	Judging rules
Green smart building evaluation *U*	Safety and durability *U*_1_	Safety *U*_11_	Reasonably improve the seismic performance of buildings
			Personnel safety protection measures
			Products or accessories with safety functions
			Antislip measures for indoor and outdoor floors
			People and vehicles are divided and the traffic system is sufficiently illuminated
		Durability *U*_12_	Improve building adaptability
			Improve the durability of building components
			Improve the durability of building structure materials
			Reasonable use of decoration building materials
	Health and comfort *U*_2_	Indoor air quality *U*_21_	Control the concentration of major indoor air pollutants
			Decoration building materials meet national standards
		Water quality *U*_22_	Direct drinking water, landscape water, and other water quality meet national standards
			Water storage facilities such as pools meet sanitary requirements
			Permanent identification of water supply and drainage pipeline equipment
		Acoustic environment and light environment *U*_23_	Optimize the indoor acoustic environment of the main room
			The main room divider has good sound performance
			Make full use of natural light
		Indoor hot and humid environment *U*_24_	Good indoor hot and humid environment
			Improve natural ventilation effect
			Improve indoor thermal comfort
	Convenience of life *U*_3_	Mobility and barrier-free *U*_31_	The site has convenient transportation
			Public areas meet all-age design requirements
		Service facilities *U*_32_	Provide convenient public services
			Open urban green spaces, squares, and other venues
			Reasonably set up fitness venues and spaces
		Property management *U*_33_	Formulate energy-saving, water-saving, and material-saving greening plans
			The average daily water consumption of the building meets the national standard
			Regularly evaluate the building operation effect
			Establish a green education publicity and practice mechanism
	Save resources *U*_4_	Land saving and land use *U*_41_	Economical and intensive use of land
			Reasonable development and utilization of underground space
			Reasonable parking design
		Energy-saving and energy utilization *U*_42_	Optimizing the thermal performance of building envelope
			Energy efficiency of heating and air conditioning system is better than national standard
			Reduce energy consumption of heating and air conditioning systems
			Adopt energy-saving equipment and energy-saving measures
			Take measures to reduce building energy consumption
			Reasonable use of renewable resources
		Water-saving and water resources utilization *U*_43_	Use higher-efficiency sanitary appliances
			Use water-saving equipment for irrigation and cooling water
			Comprehensive utilization of rainwater to make landscape water
			Use nontraditional water sources
		Wood-saving and green building materials *U*_44_	Integrated design and construction of civil engineering and decoration
			Reasonable selection of building structure materials and components
			Industrialized interior parts are selected for building decoration
			Use recyclable and reusable materials
			Choose green building materials
	Livable environment *U*_5_	Site ecology and landscape *U*_51_	Reasonable layout of buildings and landscapes
			Planning surface and roof stormwater runoff
			Reasonably set up green land
			Reasonably arrange outdoor smoking areas
			Set up green rainwater infrastructure
		Outdoor physical environment *U*_52_	The environmental noise inside the venue is better than the national standard
			Building and lighting design to avoid light pollution
			Comfortable and natural ventilation
			Reduce heat island strength
	Smart *U*_6_	Smart security *U*_61_	Equipped with public safety smart warning function
			Video surveillance with detection function
			Set up an emergency response system
			Computer room engineering and its own protective measures specification
			Effective display of video security monitoring system
			Fire and security have linkage function and work normally
			The security system has the ability to prevent damage
			The security system uses a dedicated transmission network
		Smart architecture and platform *U*_62_	Support the deployment of IoT application services
			The platform can centrally monitor and manage each subsystem
			The platform follows the principle of modular construction
			The platform supports secondary development
			Realize equipment life cycle monitoring and management
			With the docking function of smart building operation and maintenance platform
			The platform can intelligently analyze data
			Specific applications such as data sharing
		Smart operation *U*_63_	Has a smart parking management system
			Has a smart property management system
			Realize smart home with IoT technology
			With personnel positioning indoor navigation service
			Complete information query and release system
			Wireless network coverage on demand
			Access to smart platform for main electrical building equipment
			With office automation system
			Has a building energy metering management platform
			Set up an automatic remote metering system
			Set up air quality monitoring and release system
			Set up an online monitoring system for water quality and water supply and drainage
	Innovation and characteristics *U*_7_	Improvement and innovation *U*_71_	Further reduce the energy consumption of heating and air conditioning systems
			Architectural style design and inheritance of architectural culture
			Increase the green capacity of the site
			Reasonable selection of abandoned sites
			Structural system and building components meet requirements
			Apply BIM technology
			Reduce carbon emission intensity per unit area
			Green construction and management
			Use of insurance products for potential defects in construction project quality
		Characteristics *U*_72_	Obtain the green building logo
			Meet the requirements of the national grid
			Set equipment monitoring health index
			Meet the individual needs of different acquaintances
			Apply big data artificial intelligence and other technologies

Remarks: Innovation and characteristics are the corresponding improvement of *U*1–*U*6 index factors.

**Table 2:** Weights of comprehensive evaluation indicators of green smart building project.

Target layer	First-level indicators	First-level weight	Secondary indicators	Secondary weight	Weights
*U*	*U*_*i*_	*W*_*i*_	*U*_*ij*_	*W*_*j*_	*W*_*ij*_ = *W*_*i*_^*∗*^*W*_*j*_
Green smart building evaluation system *U*	Safety and durability *U*_1_	0.1481	Safety *U*_11_	0.7500	0.1111
			Durability *U*_12_	0.2500	0.0370
	Health and comfort *U*_2_	0.0607	Indoor air quality *U*_21_	0.1178	0.0072
			Water quality *U*_22_	0.0550	0.0033
			Acoustic environment and light environment *U*_23_	0.2634	0.0160
			Indoor hot and humid environment *U*_24_	0.5638	0.0342
	Convenience of life *U*_3_	0.0253	Mobility and barrier-free *U*_31_	0.1047	0.0026
			Service facilities *U*_32_	0.6370	0.0161
			Property management *U*_33_	0.2583	0.0065
	Save resources *U*_4_	0.3451	Land saving and land use *U*_41_	0.0550	0.0190
			Energy-saving and energy utilization *U*_42_	0.5638	0.1946
			Water-saving and water resources utilization *U*_43_	0.2634	0.0909
			Wood-saving and green building materials *U*_44_	0.1178	0.0407
	Livable environment *U*_5_	0.0607	Site ecology and landscape *U*_51_	0.7500	0.0455
			Outdoor physical environment *U*_52_	0.2500	0.0152
	Smart *U*_6_	0.3451	Smart security *U*_61_	0.1047	0.0361
			Smart architecture and platform *U*_62_	0.2583	0.0891
			Smart operation *U*_63_	0.6370	0.2198
	Innovation and characteristics *U*_7_	0.0151	Improvement and innovation *U*_71_	0.5000	0.0076
			Characteristics *U*_72_	0.5000	0.0076

## Data Availability

The data used to support the findings of this study are included within the article.
